# Reduced current density, partially rescued by mexiletine, and depolarizing shift in activation of SCN5A W374G channels as a cause of severe form of Brugada syndrome

**DOI:** 10.1111/anec.12828

**Published:** 2021-01-19

**Authors:** Tadashi Nakajima, Tommy Dharmawan, Reika Kawabata‐Iwakawa, Shuntaro Tamura, Hiroshi Hasegawa, Takashi Kobari, Masaki Ota, Shoichi Tange, Masahiko Nishiyama, Yoshiaki Kaneko, Masahiko Kurabayashi

**Affiliations:** ^1^ Department of Cardiovascular Medicine Gunma University Graduate School of Medicine Maebashi Japan; ^2^ Division of Integrated Oncology Research Gunma University Initiative for Advanced Research Maebashi Japan; ^3^ Department of Cardiovascular Medicine National Hospital Organization Takasaki General Medical Center Takasaki Japan; ^4^ Department of Cardiovascular Medicine Japanese Red Cross Maebashi Hospital Maebashi Japan; ^5^ Gunma University Maebashi Japan

**Keywords:** Brugada syndrome, mexiletine, rescue, *SCN5A*, trafficking defect

## Abstract

**Background:**

*SCN5A*‐related Brugada syndrome (BrS) can be caused by multiple mechanisms including trafficking defects and altered channel gating properties. Most *SCN5A* mutations at pore region cause trafficking defects, and some of them can be rescued by mexiletine (MEX).

**Objective:**

We recently encountered symptomatic siblings with BrS and sought to identify a responsible mutation and reveal its biophysical defects.

**Methods:**

Target panel sequencing was performed. Wild‐type (WT) or identified mutant SCN5A was transfected into tsA201 cells. After incubation of transfected cells with or without 0.1 mM MEX for 24–36 hr, whole‐cell sodium currents (I_Na_) were recorded using patch‐clamp techniques.

**Results:**

The proband was 29‐year‐old male who experienced cardiopulmonary arrest. Later, his 36‐year‐old sister, who had been suffering from recurrent episodes of syncope since 12 years, was diagnosed with BrS. An *SCN5A* W374G mutation, located at pore region of domain 1 (D1 pore), was identified in both. The peak density of W374G‐I_Na_ was markedly reduced (WT: 521 ± 38 pA/pF, W374G: 60 ± 10 pA/pF, *p* < .01), and steady‐state activation (SSA) was shifted to depolarizing potentials compared with WT‐I_Na_ (V_1/2_‐WT: −39.1 ± 0.8 mV, W374G: −30.9 ± 1.1 mV, *p* < .01). Incubation of W374G‐transfected cells with MEX (W374G‐MEX) increased I_Na_ density, but it was still reduced compared with WT‐I_Na_ (W374G‐MEX: 174 ± 19 pA/pF, *p* < .01 versus W374G, *p* < .01 versus WT). The SSA of W374G‐MEX‐I_Na_ was comparable to W374G‐I_Na_ (V_1/2_‐W374G‐MEX: −31.6 ± 0.7 mV, P = NS).

**Conclusions:**

Reduced current density, possibly due to a trafficking defect, and depolarizing shift in activation of *SCN5A* W374G are underlying biophysical defects in this severe form of BrS. Trafficking defects of *SCN5A* mutations at D1 pore may be commonly rescued by MEX.

## 1 INTRODUCTION

Brugada syndrome (BrS) is characterized by coved‐type ST‐segment elevations in the right precordial leads in an electrocardiogram (ECG), which has a tendency to cause life‐threatening ventricular tachyarrhythmias leading to syncope or sudden death (Brugada & Brugada, 1992). Recent advances in molecular genetics have identified more than 10 responsible or related genes for BrS (Nakajima et al., 2015). *SCN5A*, which encodes the α‐subunit of cardiac voltage‐gated sodium channels (Nav1.5 or I_Na_), is the major responsible gene, and *SCN5A* mutations account for approximately 20% BrS cases (Nakajima, Kaneko, & Kurabayashi, 2015). A loss of function of I_Na_ by *SCN5A* mutations is theoretically associated with BrS, and it can be caused by multiple mechanisms including trafficking defects and altered gating properties (Antzelevitch & Yan, 2000; Nakajima, Kaneko, Saito, et al., 2015).

*SCN5A* consists of four nonidentical similar domains (DⅠ‐DⅣ), and each domain contains voltage sensor domain, segment 1 (S1)‐S4, and pore domain (PD), S5 pore loop S6 (Jiang et al., 2020; Pan et al., 2018). Although BrS‐related *SCN5A* mutations spread throughout the whole structure, combination of structural and functional studies has revealed that some *SCN5A* mutations in specific regions can be associated with specific biophysical defects (Dharmawan et al., 2019; Nakajima et al., 2019; Nakajima, Kaneko, Saito, et al., 2015).

We recently encountered two siblings with symptomatic BrS, in whom we identified an *SCN5A* W374G mutation. The mutation has already been reported as a BrS‐causing mutation (Kapplinger et al., 2010), but its biophysical defects have not been clarified. W374 position of *SCN5A* is located at pore loop in PD, close to selective filter (SF) of DⅠ (two bases apart from D372, which consists of SF), and the W374 is highly conserved among Nav family (Jiang et al., 2020), which suggested that the *SCN5A* W374G mutation was located at a structurally critical site. Intriguingly, most BrS‐related *SCN5A* mutations in the PD have been reported to be trafficking‐deficient, and some of them can be rescued by some compounds including mexiletine (MEX) (Hu et al., 2018; Moreau et al., 2012; Pfahnl et al., 2007; Shinlapawittayatorn et al., 2011; Tan et al., 2006; Valdivia et al., 2002, 2004; Zhang et al., 2015).

Therefore, we sought to reveal the biophysical defects of the *SCN5A* W374G mutation. Moreover, since it caused a reduced current density, we examined whether MEX can restore it.

## 2 METHODS

### 2.1 Genetic analysis

This study was approved by the institutional ethics review board (approval number: 2017–15). Written informed consent for the genetic and functional analyses was obtained from the subjects.

Genomic DNA was extracted from peripheral blood lymphocytes, and target panel sequencing of 72 genes, including inherited arrhythmia syndrome‐related genes, was performed as previously described (Nakajima et al., 2020). Average read depth in analyzable target region was 197 in male and 244 in female sample. The percentage of analyzable target base with at least 20 reads were 98.8 in male and 98.5 in female sample. Confirmation of nucleotide substitution was performed by Sanger sequencing. Exon 9 of *SCN5A* (NM_198056.2) was analyzed as previously described (Nakajima et al., 2011).

### 2.2 Electrophysiological experiments

Wild‐type (WT) human heart sodium channel α‐subunit cDNA subcloned into pcDNA3.1 vector (hH1‐pcDNA3.1) and a plasmid containing β‐subunit (pGFP‐IRES‐hβ1) were provided by Dr. Naomasa Makita (National Cerebral and Cardiovascular Center Research Institute). Site‐directed mutagenesis for *SCN5A* W374G was constructed using a QuikChange II Site‐Directed Mutagenesis Kit (Agilent Technologies, Santa Clara, California). The human kidney cell line tsA201 was transiently transfected using Lipofectamine 2000 with 0.5 μg of hH1‐pcDNA3.1 or 0.5 μg of mutant (W374G)‐pcDNA3.1 in combination with 0.5 μg of pGFP‐IRES‐hβ1. Transfected tsA201 cells were maintained as previously described (Dharmawan et al., 2019; Nakajima, Kaneko, Saito, et al., 2015). W374G‐transfected cells were incubated with or without 0.1 mM mexiletine hydrochloride (MEX) (Sigma‐Aldrich, Japan), as Pfahnl et al. reported (Pfahnl et al., 2007), for 26–36 hr before current recordings.

Membrane sodium currents (I_Na_) were recorded using whole‐cell patch‐clamp techniques at room temperature (23–25°C) as previously described (Dharmawan et al., 2019; Nakajima, Kaneko, Saito, et al., 2015). Briefly, the bath solution for recording membrane currents contained (in mM) 145 NaCl, 4 KCl, 1.8 CaCl_2_, 1 MgCl_2_, 10 HEPES, and 10 glucose (pH 7.35 with NaOH), and the pipette solution contained (in mM) 10 NaF, 110 CsF, 20 CsCl, 10 EGTA, and 10 HEPES (pH 7.35 with CsOH). The electrode resistances were 1.1–2.0 MΩ. Data acquisition was performed using an Axopatch 200B amplifier and pCLAMP10.3 (Molecular Devices, Sunnyvale, CA, USA). All pulse protocols are shown in each Figure.

### 2.3 Statistical analysis

All data are expressed as mean ± standard error. The unpaired Student's *t* test was used to analyze differences. P values < 0.05 were considered to be statistically significant.

## 3 RESULTS

### 3.1 Case presentation

**FIGURE 1:**
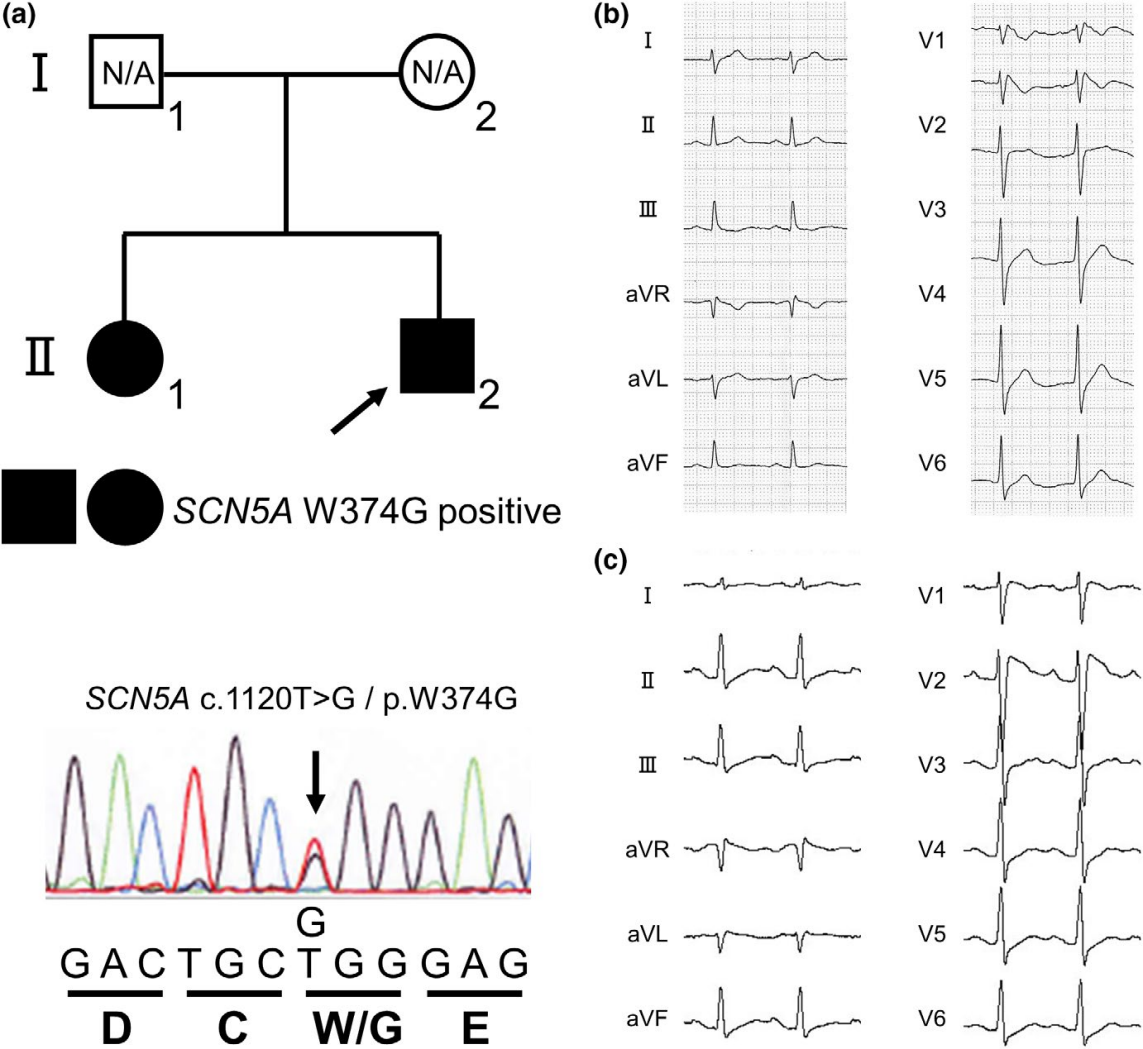
Diagnosis of Brugada syndrome and identification of an *SCN5A* W374G mutation. (A) A pedigree of the subjects (upper panel) and sequence electropherogram of the proband (lower panel). Arrow indicates the proband. N/A indicates not clinically and genetically assessed. (B) 12‐lead ECG of the proband. C. 12‐lead ECG of the proband's sister

The proband (Ⅱ‐2) (Figure 1A), a 29‐year‐old male, lost consciousness at around midnight at karaoke room, then recovered spontaneously. However, he lost consciousness again in the ambulance. His ECG monitor displayed ventricular fibrillation (VF), and then, he was resuscitated by an automated external defibrillator. His 12‐lead ECG recorded at the emergency room showed coved‐type ST elevations in V1 and V2 leads (Figure 1B); thus, he was diagnosed with BrS. Echocardiography revealed that he had no structural heart disease. His 36‐year‐old elder sister had experienced 9 episodes of syncope since 12 years old, and had been misdiagnosed with epilepsy or vasovagal syncope. However, since her younger brother (the proband) was diagnosed with BrS, she was examined by cardiologists. Her 12‐lead ECG showed coved‐type ST elevations in V1 and V2 leads (Figure 1C); thus, she was diagnosed with BrS. Both siblings were implanted with implantable cardioverter–defibrillator.

### 3.2 Identification of an *SCN5A* W374G mutation

Target panel sequencing of 72 genes including inherited arrhythmia syndrome‐related genes was performed in the proband and his elder sister as described previously (Nakajima et al., 2020). An *SCN5A* W374G mutation, which has already been reported as a BrS‐causing mutation (Kapplinger et al., 2010), was identified in both siblings, and it was validated by Sanger sequencing (Figure 1A). Unfortunately, consent for genetic analysis and participation to this study were not obtained from their parents.

### 3.3 Biophysical defects of the *SCN5A* W374G mutation

**FIGURE 2:**
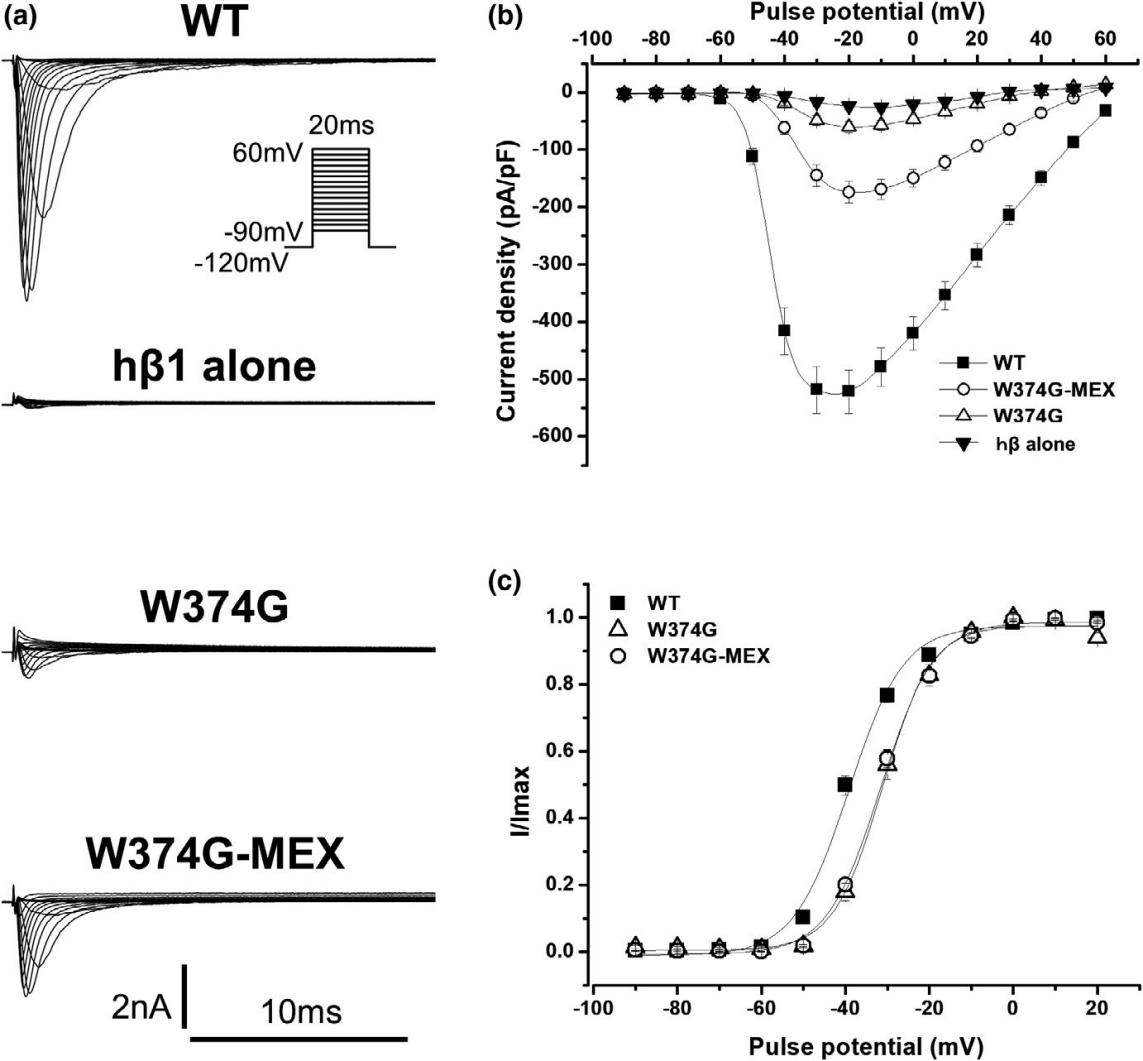
Decreased current density, partial rescue by mexiletine, and depolarizing shift of steady‐state activation of W374G‐I_Na_. (A) Representative current tracings from the cells transfected with wild‐type (WT) SCN5A plus human β1 subunit (hβ1) (WT‐I_Na_) (upper panel), those with hβ1 alone (second row panel), those with W374G SCN5A plus hβ1 (W374G‐I_Na_) (third row panel), and those with W374G SCN5A plus hβ1 after incubation with 0.1 mM mexiletine (W374G‐MEX‐I_Na_) (lower panel). (B) Current–voltage relationship of WT‐I_Na_ (filled squares, *n* = 19), hβ1 alone (filled reverse triangles, *n* = 6), W374G‐I_Na_ (open triangles, *n* = 19), and W374G‐MEX‐I_Na_ (open circles, *n* = 26). Peak currents obtained by the pulse protocol were normalized to cell capacitances. Current densities at −20 mV are shown in Table 1. C: Steady‐state activation of WT‐I_Na_ (filled squares, *n* = 19), W374G‐I_Na_ (open triangles, *n* = 10), and W374G‐MEX‐I_Na_ (open circles, *n* = 26). Plots were fitted with a Boltzmann function. Fitted data are shown in Table 1

**FIGURE 3:**
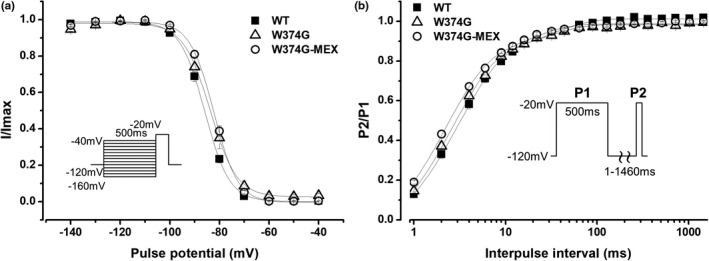
Steady‐state inactivation and recovery from inactivation of WT‐I_Na_, W374G‐I_Na_, and W374G‐MEX‐I_Na_. A: Steady‐state inactivation of WT‐I_Na_ (filled squares, *n* = 17), W374G‐I_Na_ (open triangles, *n* = 12), and W374G‐MEX‐I_Na_ (open circles, *n* = 20). Plots were fitted with a Boltzmann function. Fitted data are shown in Table 1. B: Time course of recovery from inactivation of WT‐I_Na_ (filled squares, *n* = 14), W374G‐I_Na_ (open triangles, *n* = 9), and W374G‐MEX‐I_Na_ (open circles, *n* = 12). Plots were fitted with a double exponential function. Fitted data are shown in Table 1

We transfected wild‐type (WT) SCN5A plus hβ1, W374G SCN5A plus hβ1, and hβ1 alone in tsA201 cells, and recorded whole‐cell I_Na_ (WT‐I_Na_, W374G‐I_Na_, and hβ1 alone, respectively).

hβ1 alone exhibited very small inward currents (Figure 2A), which were not different from endogenous currents (data not shown). On the other hand, W374G SCN5A plus hβ1 produced small I_Na_ (W374G‐I_Na_). The peak density of W374G‐I_Na_, measured at −20 mV from a holding potential of −120 mV, was significantly larger than that of hβ1 alone, but the peak density of W374G‐I_Na_ was significantly smaller than that of WT‐I_Na_ (Table 1) (Figure 2A,B). Since the amplitudes of W374G‐I_Na_ from some cells were too small to evaluate the gating properties properly, we analyzed cells where the peak current amplitudes were over 0.8 nA. The steady‐state activation (SSA) of W374G‐I_Na_ was significantly shifted to depolarizing potentials (~8.2 mV) in comparison with that of WT‐I_Na_ (Table 1) (Figure 2C). The steady‐state inactivation (SSI) of W374G‐I_Na_, assessed by a prepulse duration of 500 ms, was comparable to that of WT‐I_Na_. (Table 1) (Figure 3A). The recovery from inactivation of I_Na_ was assessed using a double‐pulse protocol and was fitted by a double exponential function. The fast component of recovery from inactivation of W374G‐I_Na_ was accelerated in comparison with that of WT‐I_Na_ (Table 1) (Figure 3B).

**TABLE 1:** Parameters of activation, steady‐state inactivation, and recovery from inactivation

	Activation			Steady‐state inactivation		Recovery from inactivation			
	Current density (pA/pF) at −20 mV	V_1/2_ (mV)	K (mV)	V_1/2_ (mV)	K (mV)	A fast	τ fast (ms)	A slow	τ slow (ms)
WT‐I_Na_	521 ± 38 (*n* = 19)	−39.1 ± 0.8	6.25 ± 0.33	−85.9 ± 0.6 (*n* = 17)	4.76 ± 0.13	0.83 ± 0.01 (*n* = 14)	3.11 ± 0.20	0.17 ± 0.01	34.6 ± 3.3
W374G‐I_Na_	60 ± 10^b^ (*n* = 19)	−30.9 ± 1.1^b^ (*n* = 10)	5.70 ± 0.31	−83.8 ± 1.6 (*n* = 12)	4.40 ± 0.08^a^	0.81 ± 0.03 (*n* = 9)	2.46 ± 0.22^a^	0.19 ± 0.03	30.7 ± 8.8
W374G‐MEX‐I_Na_	174 ± 19^b^, ^c^ (*n* = 26)	−31.6 ± 0.7^b^	6.01 ± 0.17	−82.4 ± 0.6^b^ (*n* = 20)	4.54 ± 0.10	0.80 ± 0.02 (*n* = 12)	2.18 ± 0.13^b^	0.20 ± 0.02	24.9 ± 6.9

Note

V_1/2_, voltage at which half of the channels are available to open; K, slope factor; A, fractional amplitude; fast, fast component; slow, slow component; τ, time constant

^a^ *p* < 0.05 versus WT

^b^ *p* < 0.01 versus WT

^c^ *p* < 0.01 versus W374G.

### 3.4 Partial rescue of W374G‐I_Na_ density by mexiletine

Mexiletine (MEX), a sodium channel blocker, has been reported to rescue some *SCN5A* trafficking‐deficient mutations or restore reduced current density of mutant I_Na_ (Hu et al., 2018; Moreau et al., 2012; Pfahnl et al., 2007; Shinlapawittayatorn et al., 2011; Tan et al., 2006; Valdivia et al., 2002, 2004; Zhang et al., 2015), while it had a small impact on the current density of WT‐I_Na_ (Hu et al., 2018; Tan et al., 2006; Valdivia et al., 2002). Therefore, we incubated W374G‐transfected cells with 0.1 mM MEX for 24–36 hr before current recordings.

The peak amplitude of W374G‐I_Na_ after incubation with MEX (W374G‐MEX‐I_Na_) was significantly larger than that of W374G‐I_Na_, but it was still significantly smaller than that of WT‐I_Na_ (Table 1) (Figure 2A,B). However, the SSA of W374G‐MEX‐I_Na_ was still comparable to that of W374G‐I_Na_, and it was significantly shifted to depolarizing potentials (~7.5 mV) in comparison with that of WT‐I_Na_ (Table 1) (Figure 2C). The fast inactivation rate of W374G‐MEX‐I_Na_, assessed by fitting inactivating currents by a single exponential function, was comparable to that of WT‐I_Na_ (data not shown). The SSI of W374G‐MEX‐I_Na_ was comparable to that of W374G‐I_Na_, but it was significantly shifted to depolarizing potentials (~3.5 mV) in comparison with that of WT‐I_Na_. (Table 1) (Figure 3A). The fast component of recovery from inactivation of W374G‐MEX‐I_Na_ was comparable to that of W374G‐I_Na_, but it was significantly accelerated in comparison with that of WT‐I_Na_ (Table 1) (Figure 3B). These gating properties of W374G‐MEX‐I_Na_ were almost comparable to those of W374G‐I_Na_.

## 4 DISCUSSION

### 4.1 Reduced current density and altered gating properties of W374G‐I_Na_

We identified an *SCN5A* W374G mutation in two siblings with symptomatic BrS. The proband (29‐year‐old male) experienced VF, and his elder sister (36‐year‐old male) had experienced recurrent episodes of syncope. Thus, they appear to be a severe form of BrS.

A loss of function of I_Na_ in BrS can be caused by multiple mechanisms including trafficking defects and altered gating properties (Antzelevitch & Yan, 2000; Nakajima, Kaneko, Saito, et al., 2015). Our functional study revealed that the *SCN5A* W374G expressed I_Na_. However, the density of W374G‐I_Na_ was very small compared with that of WT‐I_Na_. This suggested that the *SCN5A* W374G might be a trafficking‐deficient mutation. In addition, W374G‐I_Na_ displayed a large depolarizing shift (~8.2 mV) in SSA, while it also displayed a small acceleration of fast component of recovery from inactivation (tau‐WT: 3.11 ms versus W374G: 2.46 ms). In addition to a reduced current density, a large depolarizing shift in SSA was also an underlying mechanism of a loss of function of W374G‐I_Na_.

### 4.2 Trafficking‐deficient *SCN5A* mutations rescued by MEX

The expression of proteins in the cell membrane involves a series of processes including gene transcription, RNA processing, protein synthesis, assembly and post‐translational modification, transport to the cell surface, anchoring to the cytoskeleton, and regulation of endocytosis and controlled degradation of protein (Herfst et al., 2004). Most trafficking‐deficient *SCN5A* mutations are thought to misfold and retain at endoplasmic reticulum (ER); thus, their transport to Golgi apparatus is impaired.

There have been many trafficking‐deficient *SCN5A* mutations associated with BrS. Several compounds have been reported to be able to rescue a part of trafficking‐deficient *SCN5A* mutations. Notably, among trafficking‐deficient *SCN5A* mutations, most reported to be rescued by MEX are located at pore‐S6 regions in DⅠ, DⅢ, and DⅣ, but not DⅡ (Hu et al., 2018; Moreau et al., 2012; Pfahnl et al., 2007; Shinlapawittayatorn et al., 2011; Tan et al., 2006; Valdivia et al., 2002, 2004; Zhang et al., 2015), except for an *SCN5A* F1473S mutation located at the DⅢ‐DⅣ linker (Ruan et al., 2010). The fact that MEX partially rescued the *SCN5A* W374G mutation, which is located at pore loop of DⅠ, is consistent with this notion.

Regarding the gating properties, MEX could not restore the depolarization shift in SSA and acceleration of fast component of recovery from inactivation of W374G‐I_Na_.

### 4.3 Possible association between MEX binding and trafficking rescue

Residues in S6 segments of DⅠ, DⅢ, and DⅣ, but not DⅡ, have been reported to affect local anesthetic binding (Yarov‐Yarovoy et al., 2002). Recently, Nakagawa et al. reported that residues in DⅠS6, DⅢS6, and DⅣS6, but not DⅡS6, are major determinants to affect the affinity of MEX, which is supported by structural studies revealing that L1462 in DⅢS6 and F1760 in DⅣS6 face the inner pore, while N406 in DⅠS6 faces away from the pore (Nakagawa et al., 2019). Considering the fact that most *SCN5A* trafficking‐deficient mutations rescued by MEX are located at pore‐S6 regions in DⅠ, DⅢ, and DⅣ, but not DⅡ, it is conceivable that MEX binding to the pore‐S6 region of unmatured trafficking‐deficient mutant channels may stabilize the pore of the channel, then promote protein folding at ER, thereby facilitating the exit of mutant channels from the ER to Golgi apparatus. However, precise mechanisms of trafficking rescue by MEX remain to be clarified. Therefore, further studies are needed.

## 5 CONCLUSIONS

We identified an *SCN5A* W374G mutation, located at pore loop in DⅠ, in a severe form of BrS. Our functional study revealed that reduced current density, possibly due to a trafficking defect, and depolarizing shift in SSA are the underlying biophysical defects. Reduced current density of this mutation could be partially restored by MEX, which support the notion that trafficking defects of *SCN5A* mutations located at pore‐S6 regions in DⅠ, DⅢ, and DⅣ, but not DⅡ, can be rescued by MEX, and that MEX binding to the pore‐S6 regions in DⅠ, DⅢ, and DⅣ, but not DⅡ, of mutant channels is a critical step in the rescue of trafficking defects.

## CONFLICT OF INTEREST

The authors declare no conflict of interests for this article.
